# Arterial hypertension assessed “out-of-office” in a contemporary cohort of rheumatoid arthritis patients free of cardiovascular disease is characterized by high prevalence, low awareness, poor control and increased vascular damage-associated “white coat” phenomenon

**DOI:** 10.1186/ar4324

**Published:** 2013-10-02

**Authors:** Athanase D Protogerou, Demosthenis B Panagiotakos, Evangelia Zampeli, Antonis A Argyris, Katerina Arida, Giorgos D Konstantonis, Christos Pitsavos, George D Kitas, Petros P Sfikakis

**Affiliations:** 1 Rheumatology Unit and Cardiovascular Research Laboratory, First Department of Propaedeutic and Internal Medicine, Laiko Hospital, Medical School, National and Kapodistrian University of Athens, Athens, Greece; 2 Department of Nutrition and Dietetics, Group of Biostatistics, Epidemiology and Research Methods, Harokopio, University, Athens, Greece; 3 First Department of Cardiology, Ippokrateion Hospital, Medical School, National and Kapodistrian University of Athens, Athens, Greece; 4 The Dudley Group NHS Foundation Trust, Dudley, and Arthritis Research Campaign Epidemiology Unit, University of Manchester, Manchester, UK

## Abstract

**Introduction:**

Rheumatoid arthritis (RA) is associated with a high cardiovascular disease (CVD) risk, whereas arterial hypertension is a major modifiable CVD risk factor with still unclear prevalence in RA disease. We conducted a comprehensive study on hypertension characteristics evaluating for the first time out-of-office blood pressure (BP) in a typical contemporary RA cohort.

**Methods:**

Assessment of office and out-of-office BP (when office systolic/diastolic BP was >129/79) and vascular studies including evaluation of aortic stiffness, carotid hypertrophy/plaques and ankle-brachial index, were performed in 214 consecutive, consenting RA patients free of CVD (aged 58.4 ± 12.3 years, 82% women). As comparators regarding office hypertension measurements, data from 214 subjects (1:1 matched for age and gender with the RA patients) derived from a cohort designed to assess the prevalence of hypertension in the general population were used.

**Results:**

The prevalence of declared known hypertension in the RA population was 44%. Of the remaining RA patients, 2 in every 5 individuals had abnormal office BP (systolic/diastolic >139/89 mmHg), contributing to almost double the prevalence of declared/office hypertension compared to the general matched population (67% vs. 34%). Out-of-office (home or ambulatory 24 hour) BP measurements revealed that: (i) a 54% prevalence of actual hypertension in RA, in other words almost 10% of the patients were unaware of having hypertension and (ii) 29% of the RA patients with known hypertension were not well controlled. Actual hypertension was positively associated with age and body mass index, and inversely with the use of biologic drugs. Overall, almost 1 out of 5 presented the 'white coat’ phenomenon. An intermediately compromised vascular phenotype was evident in this “white coat” subgroup (lying between patients with sustained normotension and sustained hypertension) in terms of aortic stiffness, carotid hypertrophy and ankle-brachial index, even after adjustment for confounders.

**Conclusion:**

Beyond any doubt on the basis of out-of-office evaluation, arterial hypertension in RA has a high prevalence, low awareness and poor control, as well as substantial and vascular damage-associated “white coat” phenomenon. Thus, correct diagnosis and effective treatment of hypertension is of key importance in RA for CVD risk reduction.

## Introduction

Compared to the general population, patients with rheumatoid arthritis (RA) have almost twice the risk of developing cardiovascular disease (CVD) [1] and their risk for myocardial infarction corresponds to that of non-RA patients who are 10 years older [2]. CVD mortality accounts for almost half of all-cause mortality in RA [3,4]. This is thought to be mediated by accelerated atherosclerosis [5,6] and subclinical vasculitis [7,8]. The increased burden of established CVD risk factors in RA patients [9] explains only partly the excess CVD mortality [10], suggesting that systemic inflammation participates in the development of CVD.

Arterial hypertension is a major modifiable CVD risk factor worldwide [11]. Previous studies suggest that the prevalence of hypertension is increased in RA [12,13], possibly related to clinical status [14] and the related physical inactivity [15] as well as to genetic factors [16,17]. As recently reviewed, however, the evidence is still conflicting [18]. This is in part due to the fact that the existing data are based on single-visit office blood pressure (BP) readings, whereas data based on out-of-office BP evaluation (which provides the gold-standard assessment of BP-associated CVD risk [19]) are lacking. As a result, data on white coat hypertension (WCH) and masked hypertension (MH) phenomena, two BP phenotypes associated with potentially intermediate CVD risk [19], are also lacking in RA patients.

Elevated BP in RA may derive from increased oxidative stress and systemic inflammation, impaired endothelial function, vasoconstriction and increased total peripheral resistance, as well as arterial stiffening [5,9,12,13]. RA-related drugs, such as corticosteroids and nonsteroidal anti-inflammatory drugs, and particular genetic polymorphisms, together with environmental factors, may also precipitate BP elevation in RA [12,13,16,17]. In contrast, anti–tumor necrosis factor and anti–interleukin 6 biologics appear to have beneficial effects on endothelial function and arterial stiffness [20,21] and thus may reduce BP independently of Disease Activity Score [14].

In a contemporary cohort of consecutive RA patients free of established CVD, we evaluated BP and assessed vascular organ damage. Our principal hypotheses were (1) that the prevalence of hypertension in RA is higher than that in the general population and (2) that specific BP diagnostic phenotypes, such as WCH and MH phenomena, have different clinical significance in terms of vascular damage in RA patients. The following were our specific aims: (1) to compare the prevalence of abnormal office BP measurements to those observed in a 1:1 age- and gender-matched general population group free of CVD, (2) to assess the actual prevalence of hypertension in RA by applying, for the first time, out-of-office BP measurements to identify the awareness and effectiveness of BP control as well as the prevalence of resistant hypertension, (3) to identify factors associated with the presence of hypertension in RA and (4) to identify specific BP diagnostic phenotypes, such as WCH and MH phenomena, and their association with the extent of vascular damage.

## Methods

The present study was approved by the Ethical/Scientific Committee of the “Laikon” Hospital, and all participants provided their informed consent to participate according to the Declaration of Helsinki.

### Rheumatoid arthritis patients

From September 2010 until September 2012, 242 consenting, consecutive patients (mean age 59.2 ± 12.3 years, 195 women (81%)) attending the outpatient Rheumatology Clinic and meeting the American College of Rheumatology classification criteria [22] were examined at the Cardiovascular Research Laboratory for global CVD risk stratification. Ninety-five percent of them were inhabitants of the Attica province of Greece (which includes 78% urban and 22% rural areas – mainly the city of Athens and its suburbs). All patients underwent a medical interview and examination so that we could record all the known classical CVD risk factors and drug treatments as well as the presence of CVD. All biochemical data were retrieved from the patient’s records at the outpatient Rheumatology Clinic of the department. The estimated creatinine clearance (eCCL) was assessed by using the Cockcroft-Gault formula. Two hundred fourteen RA patients were included in the present analysis (mean age 58.4 ± 12.3 years, 176 women (82%)) after excluding 18 patients with established CVD, 2 with estimated creatinine clearance less than 30 ml/min (due to the possibility of secondary hypertension) and 8 patients without available office BP recordings.

### Control group

RA patients were matched 1:1 by age and sex with 214 individuals randomly derived from the ATTICA study database [23]. In brief, the ATTICA study is a general population-based health and nutrition survey that was conducted from May 2001 to December 2002 in the province of Attica, Greece. The original sample included 3,042 participants, and the data selection was performed among 2,285 (1,128 men) who were free of CVD or inflammatory disease.

### Protocols for arterial blood pressure and hypertension assessment

BP in RA patients was measured in the morning (8:30 AM to 12:30 PM) during the vascular tests after at least ten minutes of rest in the supine position under controlled room temperature (22°C to 25°C). Triple BP recording was performed (with one-minute intervals between readings) using the right arm and a previously validated automated oscillometric device (WatchBP Office; Microlife AG, Widnau, Switzerland) [24]. The average of the three BP readings was calculated and used in the subsequent analysis.

All participants with office systolic (S)BP/diastolic (D)BP 129/79 mmHg or lower were considered to have optimal office BP and thus to have low probability of exhibiting the MH phenomenon, since this is particularly observed in individuals with high normal office BP [25]. All others were advised to perform out-of-office BP measurements (either home BP monitoring (HBPM) or 24-hour ambulatory BP monitoring (ABPM) according to their own preference). These two methodologies are equally recommended by the European Hypertension Society (ESH) 2007 guidelines [19] for the detection of the WCH and MH phenomena. HBPM was performed with a previously validated automated oscillometric device (WatchBP Home, Microlife AG) [26] according to the ESH suggested protocol (seven-day BP recordings) [27]. All patients underwent a brief tutorial about how to use the BP device correctly and how to comply with the ESH recommendations for HBPM [27]. The HBPM data were downloaded and analyzed using specifically designed software (TRITON software package; Proton Labs, Athens, Greece) according to the built-in ESH recommendations [27]. ABPM measurements were performed according to the ESH 2007 guidelines [19] using a previously validated automated oscillometric device (90207 ambulatory blood pressure monitor; Spacelabs Healthcare, Snoqualmie, WA, USA) [28]. All patients were advised to perform their regular activities during ABPM.

**Figure 1:**
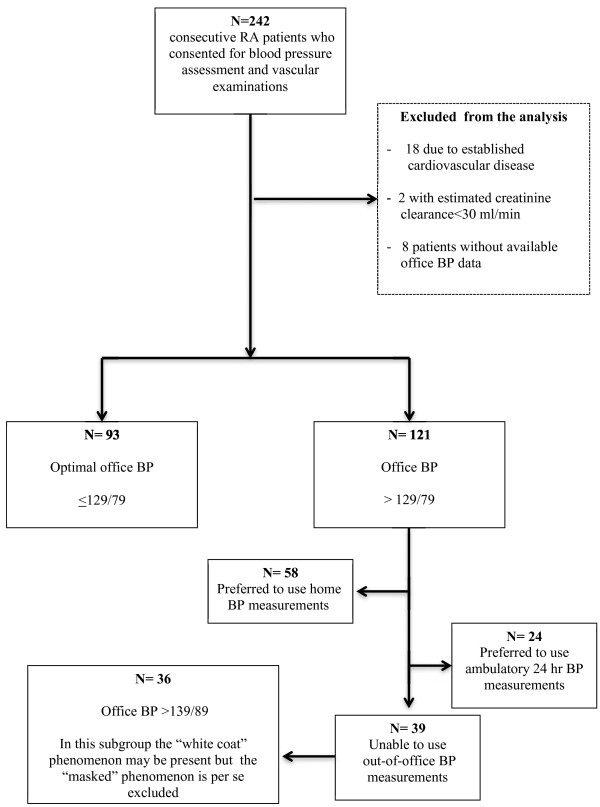
**Flow of the blood pressure assessment methods used in the rheumatoid arthritis population.** BP, blood pressure; RA, rheumatoid arthritis.

Overall, 93 of the 214 RA patients had optimal office BP readings. The data provided in the results were calculated with the assumption that RA patients with optimal office BP would have normal out-of-office BP. From among the remaining patients, 24 underwent 24-hour ABPM and 58 underwent HBPM, whereas 39 RA patients did not consent to undergo out-of-office BP assessment. Figure 1 summarizes the protocol’s flow on the basis of BP assessment methodology. These 39 patients had characteristics similar to those of the overall cohort (mean age, 62.1 ± 12.1 years; 82% women; mean body mass index, 28.9 ± 6.2 kg/m^2^; abnormal office BP, 36 patients; high normal BP, 3 patients; and hypertensives under antihypertensive drug treatment, 14) and were excluded from the analysis regarding the prevalance of WCH and MH phenomena, as well as regarding the prevalence of out-of-office hypertension (unless already under drug treatment; *n* = 14). This fact may not have led to important underestimation of the MH phenomenon, because only three of the thirty-nine patients had high normal office BP (Figure 1).

All the definitions of the hypertension phenotypes which have been applied were based on the ESH guidelines and are summarized in Table 1[19,27].

**Table 1:** **Definitions of hypertension phenotypes according to the European Society of Hypertension recommendations**^**a**^

**Characteristics**	**Definitions**
Optimal office BP	Systolic BP ≤129 and Diastolic BP ≤79
Abnormal/uncontrolled office BP	Systolic BP >139 or Diastolic BP >89
Abnormal/uncontrolled home BP	Systolic BP >134 or Diastolic BP >84
Abnormal/uncontrolled 24-hour BP	Systolic BP >129 or Diastolic BP >79
Office hypertension	Abnormal office BP and/or antihypertensive drug treatment
Out-of-office hypertension	Abnormal out-of-office BP and/or antihypertensive drug treatment
Masked hypertension phenomenon	Normal office BP in the presence of abnormal/uncontrolled out-of-office BP
White coat hypertension phenomenon	Abnormal/uncontrolled office BP in the presence of normal out-of-office BP
Sustained normal BP	Normal office BP in the presence of normal out-of-office BP
Sustained elevated BP	Abnormal/uncontrolled office BP in the presence of abnormal/uncontrolled out-of-office BP
Known hypertension	Medical interview defined status as presence of BP-lowering drug treatment or under lifestyle modification for previously diagnosed hypertension

^a^BP: blood pressure (mmHg). Definitions are based on the European Society of Hypertension Guidelines [19,27].

With regard to the control subjects, BP was measured at the end of the physical examination in participants’ workplaces or at their homes while they were in the sitting position and had been at rest for at least 30 minutes [29]. BP recordings were performed by a cardiologist three times at the right arm, which was relaxed at a 45° angle from the trunk and well-supported by a table (ELKA aneroid sphygmomanometer; Von Schlieben Co, Munich, Germany). The SBP level was determined by the first perception of sound (of tapping quality). The DBP was determined by phase V, when the repetitive sounds become fully muffled (that is, disappear). The same criteria used for RA patients were applied for BP classification.

### Vascular studies

Vascular organ damage assessment included the investigation of aortic stiffness (carotid to femoral pulse wave velocity (PWV)), common carotid hypertrophy (cross-sectional area (CSA)) and carotid atheromatosis (carotid plaques) as previously described in detail [5]. The Ankle-Brachial Index (ABI) was assessed using a validated oscillometric automated device (WatchBP Office ABI; Microlife AG) [30]. All the vascular measurements were performed by the same experienced and dedicated technician (GD Konstantonis) after BP recording. All participants were advised to abstain from any vasoactive drug or substance (including BP-lowering drugs) on the morning of the vascular examination.

### Statistical analysis

Variables were tested for normality by the Kolmogorov-Smirnov test. Paired *t*-test, χ^2^ and Wilcoxon test were applied for comparison of the 1:1 matched RA and control populations as appropriate. Binary logistic regression analysis was applied to find the independent predictors of hypertension and BP control. The classical determinants, namely, age, body mass index and sex traits, were constantly used in all models to further identify RA-related and non-RA-related parameters which were associated with the dependent variables. Regression analysis, analysis of variance and analysis of covariance (to adjust for potential confounders) were applied to detect differences between diagnostic BP phenotypes. Significance was defined as *P* < 0.05 throughout. Statistical analysis was performed using SPSS version 19.0 software (SPSS Inc, Chicago, IL, USA). The results are presented as means ± SD or percentages, and non–normally distributed continuous variables are presented as median values (50th quartile) with interquartile ranges (25th to 75th quartiles) as appropriate.

## Results

### Prevalence, awareness and control of hypertension in rheumatoid arthritis

**Figure 2:**
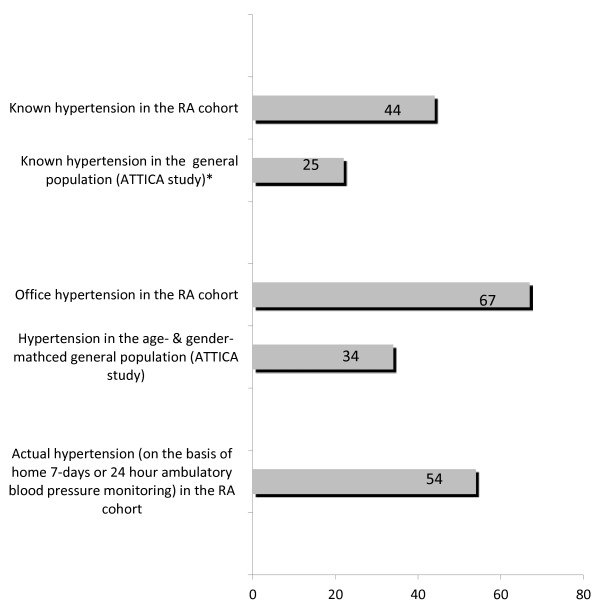
**Prevalence of blood pressure levels under the studied conditions.** Bar graph shows the prevalence in percent of known, office and actual blood pressure (BP) on the basis of out-of-office BP assessment, either seven-day home BP monitoring or twenty-four-hour ambulatory BP monitoring, in the overall rheumatoid arthritis cohort as well as in the general population of the ATTICA study.

The prevalence of known hypertension was 44% in the RA cohort. Of the remaining RA patients, almost two of every five had abnormal office BP measurements, reaching an overall rate of 67% (males: 74%, females: 66%) of office BP-defined hypertension (Figure 2). This ratio was almost double that found in the 1:1 age- and gender-matched control group (34%; males: 37%, females: 33%). RA patients had no statistically significant differences in hypercholesterolemia, diabetes mellitus, current smoking and body mass index compared to the control group from the ATTICA study (Table 2). Interestingly, although matched for age and gender, women with RA had a higher prevalence of menopause than women in the control group. RA patients had higher C-reactive protein levels than the controls (Table 2).

**Table 2:** **Demographics of the 214 patients with rheumatoid arthritis and the 1:1 age- and gender-matched control group**^**a**^

**Characteristics**	**RA**	**Control group**	***P***
Hypercholesterolemia (%)	42	28	0.176
Diabetes mellitus (%)	6	13	0.462
Current smokers (%)	30	42	0.089
Women in menopause (%)^b^	77	61	<0.001
Body mass index (kg/m^2^)	27.1 ± 5.4	25.6 ± 6.4	0.430
Total cholesterol (mg/dl)	205.6 ± 36.9	199.8 ± 40.6	0.450
Glucose (mg/dl)	93.0 ± 19.0	94.7 ± 31.10	0.419
Creatinine (mg/dl)	0.8 ± 0.2	n/a	
eCCL (ml/min)	96.0 ± 33.4	-	
C-reactive protein (mg/dl)	4.5 (2.2 to 11.4)	1.0 (0 to 3)	<0.001
Use of antihypertensive drugs (%)	44	34^c^	–
RA-related drugs (%)			
Corticosteroid (%)	70	–	
Methotrexate (%)	58	–	
Leflunomide (%)	17	–	
Cyclosporin (%)	1	–	
Nonsteroidal anti-inflammatory drugs (%)	5	–	
Biologic drugs (%)	35	–	
Hydroxychloroquine (%)	5	–	

^a^eCCL, estimated creatinine clearance using the Cockcroft-Gault formula; RA, rheumatoid arthritis. The RA patients had a mean age of 58.4 ± 12.3 years, and 82% were females. The study patients were age- and gender-matched 1:1 with a control group from the ATTICA study. Data are presented as means ± SD or medians (interquartile range) and analyzed by paired *t*-test, χ^2^ test and Wilcoxon test as appropriate. ^b^Data were available for 170 of 176 female RA subjects and 132 of 176 controls. ^c^Data are derived from a previously published paper [35].

Out-of-office BP assessment (either seven-day HBPM or twenty-four-hour ABPM) revealed that the actual prevalence of hypertension in RA was 54% (males: 55%; females: 54%). As a consequence, approximately an additional 10% of the overall population were unaware that they had hypertension (Figure 2). Moreover, 29% of the RA patients with known hypertension had uncontrolled out-of-office BP. Overall, about one of every two RA patients (52%) with known hypertension had abnormal out-of-office BP despite antihypertensive treatment.

Among the hypertensive RA patients, the prevalence of resistant hypertension (defined as uncontrolled BP in the presence of at least three antihypertensive drugs, including a diuretic) was 9% on the basis of office BP measurements or 5% on the basis of out-of-office BP readings.

### Factors associated with hypertension and uncontrolled blood pressure in rheumatoid arthritis

All the RA-related and nonrelated parameters were assessed on the basis of logistic regression analysis, with data presented as multivariate adjusted odds ratios (aOR) and 95% confidence intervals (CI). Abnormal office BP measurements were positively associated with age (aOR = 1.08; 95% CI = 1.04 to 1.2), body mass index (aOR = 1.11; 95% CI = 1.04 to 1.21), presence of menopause (aOR = 4.8; 95% CI = 1.6 to 14.3) and male gender (aOR = 3.3; 95% CI = 1.07 to 10.2). Abnormal office BP measurements were inversely associated with the use of biologic drugs (aOR = 0.40; 95% CI = 0.17 to 0.92). Exercise was marginally excluded from the model. The presence of actual (out-of-office) hypertension was positively associated only with age (aOR = 1.09; 95% CI = 1.06 to 1.36) and body mass index (aOR = 1.15; 95% CI = 1.06 to 1.25) and was inversely associated with the use of biologic drugs (aOR = 0.46; 95% CI = 0.21 to 0.99). Among the hypertensive RA patients, the use of leflunomide was the only RA-related parameter that was associated with uncontrolled out-of-office BP (aOR = 3.63; 95% CI = 1.04 to 12.65), even after adjustment for age, body mass index and gender.

### Blood pressure diagnostic phenotypes in rheumatoid arthritis and vascular damage

**Figure 3:**
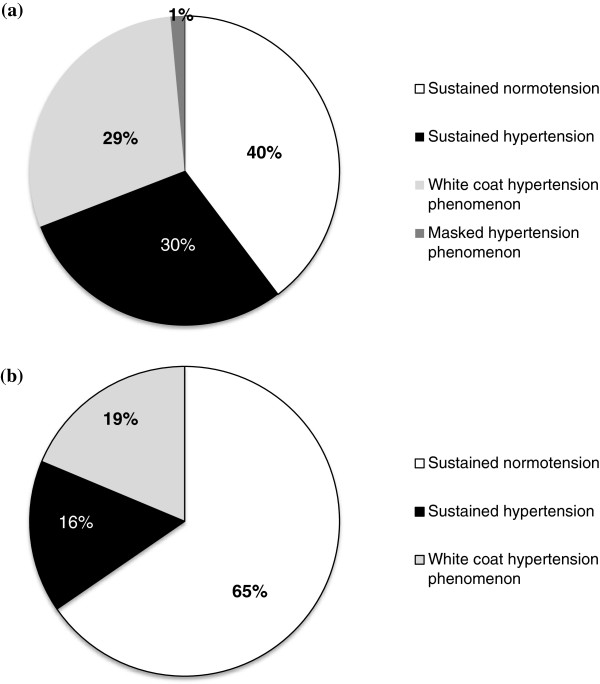
**Prevalence of white coat and masked hypertension phenomena in the rheumatoid arthritis cohort. (a)** Patients treated for hypertension. **(b)** Patients not treated for hypertension.

Less than 1% of the RA population exhibited the MH phenomenon. On the contrary, of all RA patients, almost one (21%) of every five exhibited the WCH phenomenon; that is, 29% of those treated for hypertension (Figure 3a) and 19% of those not treated for hypertension (Figure 3b) had WCH. Interestingly, these patients, regardless of the presence or not of antihypertensive treatment (data not shown), presented an intermediately compromised vascular phenotype as assessed by well-established biomarkers of arterial damage (Table 3). As shown in Table 3, WCH was associated with compromised aortic stiffness (PWV), common carotid hypertrophy (CSA), carotid plaques and ABI, all biomarkers which are related to CVD mortality in both hypertension and RA, at levels between sustained normotension and sustained hypertension, although out-of-office BP levels were comparable between the WCH and sustained normotension groups. Because significant differences in age, body mass index and smoking were found between the three groups (Table 2), we performed further adjustment for potential confounders (including age, gender, body mass index, smoking and mean BP) and found that the differences regarding PWV, right CSA and right ABI persisted between the three groups (Table 2).

**Table 3:** **Blood pressure phenotypic phenomena and vascular damage in patients with rheumatoid arthritis**^**a**^

**Characteristics**	**SN (*N *** **= 97)**	**WCH (*N *** **= 37)**	**SH (*N *** **= 40)**	***P***
BP				
Office SBP (mmHg)	115.1 ± 9.2	142.9 ± 11.1	151.8 ± 16.6	
Office DBP (mmHg)	70.9 ± 5.8	82.2 ± 7.0	86.2 ± 9.5	
Out-of-office SBP (mmHg)	123.7 ± 9.5	121.6 ± 7.3	141.2 ± 8.3	
Out-of-office DBP (mmHg)	77.6 ± 3.4	72.4 ± 68	83.1 ± 10.0	
Vascular damage				
L CCA CSA (mm^2^)	13.1 ± 3.0	14.5 ± 3.7	15.1 ± 3.2	0.002/0.251^b^
R CCA CSA (mm^2^)	12.5 ± 2.8	14.0 ± 3.8	15.2 ± 2.7	<0.001/0.027^b^
Presence of plaque (%)	49	59	72.5	0.044/0.354^b^
PWV (m/s)	7.8 ± 1.7	8.9 ± 2.7	9.8 ± 2.7	<0.001/<0.001^c^
L ABI (%)	1.23 ± 0.1	1.19 ± 0.9	1.21 ± 1.0	0.233
R ABI (%)	1.22 ± 0.1	1.14 ± 0.1	1.18 ± 0.1	0.002/<0.001^c^
CV risk factors				
Age, years	55.4 ± 12.7	59.7 ± 11.1	60.9 ± 10.8	0.026
Females (%)	86	84	70	0.067
DM (%)	6	5	5	0.923
Current smokers (%)				0.036
Current	38	16	25	
Ex-smokers	14	19	30	
Dyslipidemia (%)	18	24	30	0.328
BMI (kg/m^2^)	25.5 ± 4.8	28.1 ± 4.3	28.1 ± 5.7	0.003

^a^ABI, Ankle-Brachial Index; BMI, body mass index; BP, blood pressure; CCA, common carotid artery; CV, cardiovascular; CSA, cross-sectional surface area; DBP, diastolic blood pressure, DM, diabetes mellitus; L, left; R, right; SBP, systolic blood pressure; SH, sustained hypertension; SN, sustained normotension; WCH, white coat hypertension. ^b^Adjusted for age, gender, BMI and smoking. ^c^Adjusted for age, gender, BMI and smoking and for mean office BP. Data were analyzed by regression analysis, analysis of variance or analysis of covariance as appropriate. Data in the SN group are presented only for those patients who had high normal office BP and as a consequence were advised to measure out-of-office BP.

## Discussion

This is the first detailed study of the prevalence and characteristics of hypertension in RA patients without established CVD. It included age- and sex-matched non-CVD controls from the same geographical area and race; it utilized assessment of both office and out-of-office BP, thus enabling estimation of the actual prevalence of hypertension in RA, as well as of the WCH and MH phenomena, which are important in terms of awareness and inadequate therapy; and it incorporated assessment of vascular damage, allowing the exploration of its associations with specific hypertension phenotypes.

The novel important findings of our present study, based on out-of-office BP, are as follows. (1) The prevalence of hypertension in RA was clearly elevated (54%) compared to that in the general population (34%), and it would have been even higher if out-of-office BP assessment were available in the ATTICA study. (2) About 10% of the overall RA cohort (or one of every four RA patients with confirmed hypertension) were not aware that they had hypertension. (3) One of every two patients in the overall RA population (or one in every three RA patients with confirmed hypertension) had uncontrolled BP. (4) The prevalence of the MH phenomenon was negligible. (5) The WCH phenomenon was observed in almost one of every five RA patients overall, which should be regarded as an intermediate to high CVD risk BP phenotype in patients with RA because of the presence of accelerated atherosclerosis, thus raising questions about the optimal BP treatment strategy in this population. (6) We verified that particular RA treatment modalities, but not inflammation *per se*, are significantly associated with the presence of hypertension and interfere with BP control in addition to classical factors such as age and body mass index.

The observed high prevalence of hypertension (67%) on the basis of office BP is in accordance with previously reported data from studies in which similar office BP measurements were taken (70% in a population with a mean age 63 years [12] and 57% in a population with a mean age of 59 years [13]). Most importantly, even the actual prevalence of hypertension in RA on the basis of out-of-office BP assessment was 1.6 times that in the ATTICA study. The data derived from the ATTICA study were based on single-visit BP recordings (at the participants’ workplaces or homes) and are in general agreement with those reported in other studies in the Greek population, which were based on office BP readings [31-35]. It is thus expected that the prevalence of hypertension in the ATTICA study would be lower if based on out-of-office BP methods (either HBPM or ABPM).

A major aim of the present study was to compare the prevalence of hypertension in a typical contemporary cohort of RA patients to the prevalence of hypertension in the general population. Therefore, among all the relevant previously published Greek studies, we decided to use the most contemporary one in the general population and the one which was carried out in exactly the same region as the RA cohort (Attica, Athens). The ATTICA study was performed almost 10 years before the time the data from the present RA cohort were gathered and thus may not depict the updated prevalence of hypertension in the current general population, but the results are in line with all other epidemiological studies published before or since [31-35]. The fact that the precise method of BP measurement differed between the RA and non-RA cohorts may have influenced the results. However, we believe that the magnitude of the difference (twofold) observed in the prevalence of office hypertension between the RA and general populations in this study is extremely unlikely to be due to these limitations. As such, we suggest that the evidence for a higher prevalence of hypertension in RA compared to the general population can be considered conclusive.

The reason for the increased prevalence of hypertension in RA is not clear. In the present study, and in agreement with two previous studies [12,13], a direct association between the prevalence of hypertension and inflammation (as assessed by C-reactive protein) was not identified. However, we cannot exclude that systemic inflammation plays a role in the development of hypertension in RA, because (1) RA inflammation fluctuates, and this cross-sectional marker may not capture the long-term cumulative inflammatory burden, and (2) the studied population was well-controlled with conventional and biologic drugs, which affect C-reactive protein, endothelial function, arterial stiffness and BP levels [14,20,21]. Moreover, in the present study, the prevalence of menopause was increased by 15% in RA compared with the matched general female population and was independently associated with office hypertension. Given the fact that early menopause has been associated with increased prevalence of hypertension [36], as well as with the incidence of RA [37], these findings may represent a pathogenic link between hypertension and RA. However, biologic drugs exert a beneficial effect on endothelial function and arterial stiffness [20,21] and thus may prevent the incidence of hypertension [14].

The need to improve the awareness of hypertension among hypertensive RA patients is highlighted by the present study, in which the actual percentage of unawareness was 23% based on out-of-office BP measurements. Data based on out-of-office BP measurement are lacking in large general population groups. For purposes of comparison, we report herein the corresponding data from the present RA cohort on the basis of office BP measurements. Using these measurements, 46% of RA patients with hypertension were not aware that they had it, whereas the published data from the ATTICA study in a similar age group (55 to 65 years old) were about 40% [35]. In a previous study, about 35% of a RA cohort in the same age group (55 to 65 years old) who were unaware of their hypertension [12].

Moreover, the present data emphasize the need to improve the effectiveness of BP treatment in RA because 29% (on the basis of out-of-office BP) or 35% (on the basis of office BP) of those who were aware of having hypertension had uncontrolled BP. The published data from the ATTICA study in a similar age group (55 to 65 years old) were about 34% [35]. In a previous RA cohort in the same age group (55 to 65 years old), this figure was about 55% [12]. Physicians taking care of RA patients should optimize treatment control by taking into consideration the fact that leflunomide, as shown in the present study and as previously described [38], restricts effective BP control. Although the underlying mechanism is not clear, it is suggested that it might be mediated by the activation of the sympathetic nervous system [38]. Previous studies [12,13] have not provided consistent results regarding other RA-related drugs (for example, corticosteroids, nonsteroidal anti-inflammatory drugs). Similarly, in the present study, we did not identify the well-described effect of corticosteroids [39] and nonsteroidal anti-inflammatory drugs on BP. This is most probably related to treatment preferences that prevail in each cohort. In the present study, only 5% of patients were being treated with nonsteroidal anti-inflammatory drugs and only low-dose corticosteroids (less than 5 to 7 mg/day) were used. The latter may have undetectable effects on BP level as previously discussed [12]; however, their effects may be deleterious for the arteries in the long run, and thus their use should be carefully considered [40]. Notably, in the present study, body mass index was associated with the presence of hypertension as previously observed [12,13], as well as with poor BP control. Therefore, the described paradoxical association of obesity with decreased CVD in RA patients [9] seems to be mediated by other pathways that counterbalance the effect of obesity on BP.

Data regarding the prevalence of resistant hypertension in RA are lacking so far. In this cohort, the prevalence was found to be relatively high (9% in RA vs. 5% in the general population as published in the literature on the basis of office BP readings) [41]. However, this issue needs further thorough investigation because significant differences exist regarding the prevalence of resistant hypertension from population to population [41]. Further research is also needed to elucidate whether poor BP control in RA is due to the well-described worldwide physician’s inertia [42,43] or whether RA-specific peculiarities, such as treatment modalities, increase the prevalence of resistant hypertension and/or the presence of increased aortic stiffness [5,44], also contribute.

The optimal BP diagnostic method and the goals of treatment are unresolved issues in hypertension research [19]. The results of our present study suggest that there are peculiarities in the RA population because of the negligible prevalence of MH (less than 1% vs. the anticipated 10% to 15% in the general population [19]), whereas the prevalence of the WCH phenomenon was relatively high (21% vs. the anticipated 15% in the general population [19]). High prevalence of the WCH phenomenon has also been described in diabetes mellitus type 2 [45], which shares common characteristics with RA [5], including increased arterial stiffness, which may partly contribute to the WCH phenomenon. The current hypertension guidelines advise clinicians not to treat individuals who exhibit the WCH phenomenon unless there is evidence of target organ damage [19]. In the present cohort, RA patients with the WCH phenomenon (treated or not treated for hypertension) presented with substantial vascular damage. The prevalence of carotid plaques was 60%, and we observed an intermediate level of carotid hypertrophy, aortic stiffness and reduced ABI compared to those RA patients with sustained normotension and sustained hypertension. These findings are in line with previous findings in non-RA populations [19]. We suggest that, in similar cases, the decision regarding office BP reduction in RA patients by either drug treatment titration (in those already receiving antihypertensive drugs) or drug treatment initiation (in those currently untreated with drugs) should be carefully weighed on the basis of patient age, the degree of vascular damage, the presence of orthostatic hypotension and, certainly, the out-of-office BP level. This topic requires further research, given the fact that target organ damage has quite a high prevalence in RA and is closely related to hypertension [46].

## Conclusions

Physicians involved in the management of RA patients should be alert to the high prevalence and low awareness of hypertension on the basis of the present robust out-of-office BP data. They should also take into consideration the facts that specific RA treatment modalities have effects on the management of arterial hypertension and that the high prevalence of the WCH phenomenon is associated with increased CVD risk; thus these patients require close monitoring at the least. Hypertension is definitely a major contributor to the well-established high CVD risk observed in RA patients. As suggested by the recent recommendations of the European League Against Rheumatism [47], hypertension should be placed at the top of the research agenda for the reduction of CVD risk in RA, and future clinical trials should incorporate out-of-office BP assessment in their design.

## Abbreviations

ABI: Ankle-brachial index; ABPM: Ambulatory blood pressure monitoring; ANCOVA: Analysis of covariance; ANOVA: Analysis of variance; BP: Blood pressure; CSA: Cross-sectional area; CVD: Cardiovascular disease; DBP: Diastolic blood pressure; ESH: European society of hypertension; HBPM: Home blood pressure monitoring; MH: Masked hypertension; PWV: Pulse wave velocity; RA: Rheumatoid arthritis; SBP: Systolic blood pressure; SD: Standard deviation; WCH: White coat hypertension.

## Competing interests

The authors declare that they have no competing interests.

## Authors’ contributions

ADP made substantial contributions to the conception and design of the study, as well as to the acquisition, analysis and interpretation of the data, and was involved in drafting the manuscript and revising it critically. DBP made substantial contributions to the conception and design of the study, acquired data and was involved in revising the manuscript critically. EZ acquired data and was involved in revising the manuscript. KA, AAA and GD Konstantonis acquired data and were involved in critically revising the manuscript. CP made substantial contributions to the conception and design of the study and was involved in critically revising the manuscript. GD Kitas made substantial contributions to the conception and design of the study and to the interpretation of data and was involved in drafting the manuscript and revising it critically. PPS made substantial contributions to the conception and design of the study and to the interpretation of data and was involved in drafting the manuscript and revising it critically. All authors gave their approval of the final version of the manuscript for publication.
